# East and West

**Published:** 1887-10-08

**Authors:** Leslie Keith

**Affiliations:** Author of "The Chilcotes," "Uncle Bob's Niece," etc.


*(To be continued.)*

### East and West.

By Leslie Keith,

Author of "The Chilcotes," "Uncle Bob's Niece," etc.

*(Continued from p. 15.)*

### Chapter II.—David and Mary Jardine.

When David Jardine made up his mind to woo fortune in London, it does not necessarily follow that he did so because he had failed to "howk" a livelihood from his native soil. Indeed, though that was undoubtedly the case, there is no pretence at logical sequence, because, though it was quite certain he had to leave Logan-lea, it is beyond dispute that he might have willed to try his luck in Edinburgh or Glasgow. Being a Scot, however, he set his face towards the South, and added one more to that great army that has so subtly revenged the unforgotten disaster of Flodden Field.

A "sticket" shepherd, a poet, a dreamer—and—London! The conjunction has its humorous aspect, if it is not wholly melancholy. Was he thinking of Grub Street, this slim, bright-eyed young fellow, as he stood facing the green silence of the hills—of Grub Street, and the honours to be culled there—of the garret where Fame, trumpet in hand, might one day knock at the door?

"It's a stey brae," said the schoolmaster, who had descended the hill to have a last tussle with the wayward David, "and there's nothing but confusion at the end of it. Bide where ye are, among kenn't folks, my man; ye may travel further and fare waur."

Proverbial philosophy, on which the schoolmaster mainly relied, did not, however, move David an inch from his decision. The desire for a larger, fuller, completer life had seized him in a relentless grasp; it demanded to be satisfied, and it could find its fullest satisfaction nowhere save in London—the mighty, throbbing heart of the world. What highly-coloured moments, what vivid experiences, awaited him there! Cruel London, since Chatterton flung himself upon your charity, and found nothing but a grave, how many high hopes have you wrecked, how many fair dreams have you shattered! David was no "marvellous boy"; at best he had a linnet's note, too modest to be heard in the great chorus; but one does not need to be a genius to have unsatisfied longings, and the desire "for an epic life" sometimes visits quite humble and unimportant folks.

The schoolmaster, divided between anger and regret, spoke of the vast, unpitying world against which poets hurl themselves, and are often bruised and sometimes picked up in little pieces; but he might as well have expected the hills to listen and respond. When a young man has the London fever in his blood the wisdom of Solomon is foolishness in his ears. And what a world it was for a poet to turn his back on! The great green hills rising shoulder above shoulder, austere, almost solemn in their loneliness, no sound invading their silence but the cry of some wild bird or the bleat of a sheep, and these only serving to deepen it; the burns slipping noiseless down the slopes, a mere streak and flash of silver; the wide, brooding sky—here was stuff enough for a young fancy to build its "Palace of Art" out of. And for a deeper note there are the winter storms when the wind roars down the corries, and with wild sobs and shrieks whirls in mad revel round the grey, stark summits, and by-and-bye dies away in long, wailing notes that meet the ear like the despair of some ghost that dares not face the dawning. Here were subjects and backgrounds enough to set up twenty poets for life, and this small singer must needs relinquish them all!

It was this world—strangely and sadly gentle in its spring peace—that David was facing; behind him lay the little homestead, tucked in a green fold, a hardy hazel or two, twisted fantastically like rheumatic cripples by the winter rigours, keeping it company. The pallid face of the house, "begritten" with many a lashing rain, had all its narrow windows and its door set towards the hills; the faint green of the young year swept up to its threshold, except for the arrest of a stone dyke that fenced a kail-yard, wrested from the hillside and flourishing dubiously.

Everything that David handled, indeed, had a knack of ceasing to flourish, of faltering, and of finally giving up the struggle. His sheep seemed to know that he was a poet before he was a shepherd, and showed their sense of indignity by dying incontinently; the very collie was in the secret, and wore an air of dejected shame. The schoolmaster knew it too, and so did Mary, David's sister, and that was possibly the reason why, when she stepped out of the house as she now did, and joined the two men, she said to the schoolmaster—

"I am going with David."

"She will go," said David eagerly, as if in self-defence. "I've told her she'd be better at home."

"Where would I be better than with you, David?" said the girl, laying her hand on his shoulder. "There's just you and me in the world; where you are that's home to me."

There was something in these words that made the schoolmaster frown. People frown from a good many different causes, but assuredly the reason here was not because Mary was not fair to see; possibly it was because she was so very good to see, that he thought it a shame that she should be wasted on London.

It was such a face as you do not often see in London, or, indeed, in any town;—round and comely and singularly peaceful, with some of the deep quiet of her own hills in it. For the rest, she might be pretty or not—on the whole I incline to think she was not—but it matters the less, since that was never the first thing you thought of in looking at her. Here was a girl whom you might trust without hazard, who would do her best to answer any appeal you might make, and would help you so quietly and unostentatiously that you might possibly never discover the value of her services at all. Perhaps it was of these things the schoolmaster thought when he looked from the brother to the sister. She was of the order that is born to serve, while David appeared to have been created to meet her demand for self-sacrifice. It is a perfectly right and proper arrangement. If everybody had a passion for yielding and giving up to his neighbour, the world would presently come to a standstill, since there would be nothing for the policemen or lawyers or clergymen to do. There is, however, no immediate fear of this catastrophe.

The schoolmaster made a final appeal.

"Think a bit, Mary, lass," he said, "if once you go, it's for gude and a'."

"I know it," she said quietly.

"We'll come back," said David, turning round, and speaking with a gesture of confidence, "when I've made a name; when perhaps you won't be so ashamed of your old pupil."

"It's a long road that leads to fame," said the master dryly.

"We'll come back before you've had time to forget us," amended Mary, who had the art that sweet women possess of saying the right thing at the right moment; "we don't want to be forgotten—David and me."

"I mean to be remembered," said David with that youthful audacity and confidence that the old find so melancholy. He meant to be remembered by his works—his immortal works. The schoolmaster groaned as he went up the hill. "Fools, fools," he said, echoing the criticism of another of his craft who took on the whole a contemptuous view of brother man.

David had a big majority on his side. Every young man who leaves his native village to fight the world hopes to make a lasting bustle there, and to be remembered when others are forgotten. Scotland's sweetest singer, to be sure, was a follower of the plough, but was David likely to be an immortal?

David's village could wait without impatience for his poetical development, but it was not backward in criticism of his farming. That was wholly to be condemned, and David's lapses were not to be forgiven. First, he had let the bit of land down in the warm valley, where in those happier days the crops might be counted on to yield a modest profit, slip through his feckless fingers, and now the little sheep-farm on the hill-side was to go too. With their instinctive love of the soil, his fellow-villagers despised a man who could so carelessly forfeit his birthright, even for a niche in the temple of fame.

David despised them in turn, and it was left to Mary to say all the conciliatory things, and to maintain a difficult peace. Nobody saw any jest in the young shepherd's march on Olympus, but that was scarcely to be expected. Sydney Smith settled the question of Scottish humour, once for all, a long time ago. He demonstrated quite satisfactorily to himself that it does not and cannot exist, and no Scotchman, whatever his private opinion, has been bold enough to contradict him. So nobody laughed, though everybody spoke his or her mind with a most engaging frankness.

When the schoolmaster reached his own cottage, perched on a high arm of the hill, a thought visited him that immediately sent him downwards again. He lifted the latch of the farmhouse door, and walked in unceremoniously on the brother and sister. Mary was busy with small household cares, as if she would forbid herself time to think. David sat at a table drawn up close to one of the windows, absorbed in the study of certain papers spread over it. The papers were covered with irregular lines written in a cramped, uneasy hand, and occupying one side of each sheet only. David had learned somehow that this is the first of editorial demands; and though to meet it seemed a waste of paper where there was so much to say, it was as well to obey.

The schoolmaster looked down on the scattered sheets with something that went near to sarcasm in his grey eye.

"David," he said, "listen to me."

The poet looked up absently.

"Well?" he questioned impatiently, as his recognition of interruption in the shape of the schoolmaster grew: "you needn't try to turn my purpose," he said with a fretful contraction of the sensitive brows, "it's hopeless."

"Ye needn't fear," said the old man with unveiled contempt. "I'm auld enough to ken that it's wasted breath that's spent on a fool."

David's eyes flashed. No poet likes to be called names.

"Aye, my lad, it lies wi' you to gie me reason to change my opeenion."

"If that's what you came to say—"

"Man, ye would gar a saint say it!" cried the old man, in sudden explosion. "I came down the brae to have a word with ye upon another matter. Ye can do something to mend this dowie business; ye'll go straight to your relations in London?"

"No," said David shortly; "I want none of their patronage."

"You'll write to say ye're coming?"

"No, I will not."

"Ye'll let Mary write?"

"That's as she pleases. I want none of their favours. When they want to know me they can find me out. I won't seek them."

"Very well," said the man of books, "that's all I want with ye. Go back to your bit papers, lad."

David did as he was bid. He was something of a prig, this poor David; but young poets are apt to begin that way. His old friend had a kind of wrathful scorn for his pupil that strove with the love in his heart. He went out of the farm-kitchen, taking his sore to be healed by Mary.

Mary stood among the cabbages in the little garden, looking straight before her, but with no vision for the hills, now grown a deep purple under the dusky sky—rather with a striving to realise that other world, whose echoes had fallen so faintly on her green silence that she knew nothing either of its fierce joys or its fiercer pains. Beyond those shadowed summits lay the untried and the unknown. She looked up at the sound of his footfall, and summoned a smile for him. Perhaps her heart was sore too; and, being patriarchal and wise, he knew it. The collie had crept out and stood at her side, keeping vigil with her. She let her hand fall with a caress on the dog's head.

"You'll speak a word now and then to Jed?" she said; "he'll be lonesome wanting us."

"He's a fell beast," he said. He glanced at the dog, who knew well enough that he was the subject of their talk, and answered them with the eloquence of eyes and tail.

Then, suddenly, her companion looked at the girl. "You'll be lonesome too, lass?"

"Whyles," she answered simply.

The schoolmaster's light burned late that night, as he sat laboriously penning a letter. Though it was his grim fancy to live remote from men and high above his scholars on a hill-top, and though he clung with loyalty in his speech to the rough tongue of his boyhood, he could command quite a Johnsonian flow of language when he willed. It pleased him to-night to out-Johnson the Doctor in the dignified phrasing of the statement which was addressed to Charles Frankland, Esq., of Park Lane, London, and in which were set forth the claims of David and Mary Jardine to his notice. It was a ponderous and a lengthy document, and was quite as convincingly obscure as if it had emanated from the Heralds' Office.

The grim old schoolmaster knew something of human nature, and he was aware that fine ladies and gentlemen who live in Park Lane are apt to take refuge on the topmost bough of their genealogical tree at the first hint of a poor relation. David and Mary had a right to a seat on a lowest branch of the family trunk, however, and he made this as plain as the use of a great many "long-nebbit" words could do.

He gave this paper, duly sealed and circumscribed, into Mary's sacred charge, having a fine native distrust of the post, instructing her to place it in the keeping of a safe messenger on her arrival in London.

And for London the pair presently set out. Mary took a silent farewell of all her little household gods. There was an old chair—once her mother's seat—that she kissed, but she did it furtively, lest David should laugh. David had a care for nothing but those papers that were to be the passport to fame and fortune.

So in the youth of the year, the season of hope, they turned their backs on the hills, and went forth to the great battle-field.

How many men and women, boys and girls, no better equipped for the fight than these two foolish young things, does every day that breaks on London see pressing forward to tread her pavements, sure of victory, of honour, joy, and delight there? One can read it in their faces, in the hope that lives there, in the curious, innocent wonder of their glance. Alas! alas! too often does nightfall bring disenchantment.

